# Cyanoacrylate fuming method for detection of latent fingermarks: a review

**DOI:** 10.1186/s41935-017-0009-7

**Published:** 2017-07-18

**Authors:** Gurvinder Singh Bumbrah

**Affiliations:** Department of Forensic Science, Punjabi University, Patiala, 147002 India

**Keywords:** Fingermarks, Latent impressions, Cyanoacrylate fuming, Super glue, Non-porous item

## Abstract

Cyanoacrylate, also called super glue, fuming is a chemical method for the detection of latent fingermarks on non-porous surfaces such as glass, plastic etc. The method relies on the deposition of polymerized cyanoacrylate ester on residues of latent fingermarks. The method develops clear, stable, white colored fingerprints. However, several post-treatement procedures can be used to improve the contrast of developed prints. In addition to it, some pre-treatment procedures can also be used to develop aged latent fingermarks. It is an efficient, non-destrcutive and excellent procedure for developing latent fingermarks.

## Background

Fingerprints are one of the most valuable evidence due to their uniqueness. They are found on objects present at crime scene and are used to identify the suspect or criminal or link them to crime scene and weapon or object. Fingermarks are formed by sweat released from pores present on friction ridge skin of hands. Finger ridges contain large number of sweat pores. When the finger touches any surface, the sweat from these pores gets deposited in form of contours which are the mirror image of the ridge patterns. Since sweat is colorless in nature, its deposition on surface also produces colorless impressions and these impressions are called latent fingerprints (Thomas 1978; Ramotowski 2012).

Three types of glands: eccrine, apocrine and sebaceous glands are responsible for natural secretions from fingertips. Eccrine glands are widely distributed throughout the body and are particularly numerous on the palms of hands and the soles of feet. These glands produce sweat that is more than 98% water. These glands secrete chemicals as a result of general metabolism and catabolism (Knowles 1978; Kuno 1956). Latent fingerprint residues consists of secretions of the eccrine (sweat), sebaceous and apocrine glands. Sweat contains water (>98%), minerals (0.5%) and organic compounds (0.5%). Eccrine sweat consists of proteins, urea, amino acids, uric acid, lactic acid, sugars, creatinine, choline while sebaceous sweat consists of glycerides, fatty acids, wax esters, squalene and sterol esters (Scruton et al. 1975). Different kinds of optical, physical and/or chemical methods are routinely used to visualize latent fingerprints. These methods can be used alone or in combination with others to enhance the visibility of developed prints. Selection of method depends on nature (porous, semi-porous, non-porous), color and condition (wet or dry) of surface (Ramotowski 2012).

Cyanoacrylate fuming is a chemical method for the detection of latent fingermarks on non-porous surfaces such as plastic, glass, rubber bands, finished and unfinished wood etc. The method relies on the deposition of polymerized cyanoacrylate ester on latent fingermark residue (Ramotowski 2012). In 1978, Criminal Identification Division of the Japanese National Police Agency devised this method (Carrick 1983). L.W. Wood, in May of 1979, at the Northampton Police Headquarters in the United Kingdom, and, Louis Bourdon, in July of 1980, of Ontario, Canada also concurrent discovered this method (German 2003).

## The reagent

**Fig. 1:**
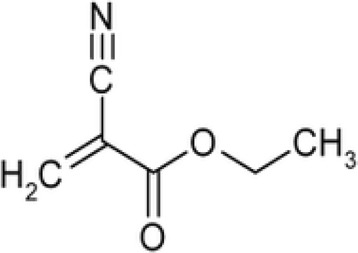
Chemical structure of Ethyl Cyanoacrylate

**Fig. 2:**
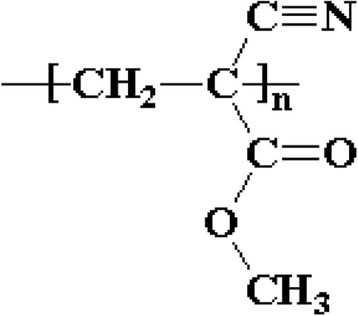
Chemical structure of Polymerized Ethyl Cyanoacrylate

**Fig. 3:**
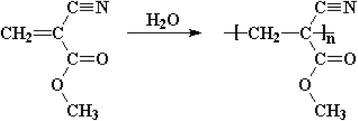
Polymerization of Ethyl Cyanoacrylate in the presence of water (weak base)

Cyanoacrylate is one type of acrylate resin. Cyanoacrylate esters, such as ethyl ester, are colorless, monomeric liquids. Alkyl 2-cyanoacrylate is acrylate ester which has the triple-bonded carbon-nitrogen (cyano or nitrile) group bonded within an ester. It forms vapors which interacts with certain eccrine components of latent fingermark residues and gets polymerized and imparts a white color to them. This hard, white polymer is known as polycyanoacrylate (Ramotowski 2012). Figures 1 and 2 present the chemical structures of ethyl cyanoacrylate and polymerized ethyl cyanoacrylate. The chemistry of cyanoacrylate polymerization process is well known (Woods et al. 1989). It is essentially an anionic polymerization that is initiated by a variety of basic compounds. The cyanoacrylate monomers possess electronegative groups which function through strong inductive effects. Strong electromeric (−E) effects of both the nitrile (−CN) and alkoxycarbonyl (−COOR) groups is responsible for the sensitivity of the alkyl-2-cyanoacrylates to weak bases. The pure monomeric cyanoacrylates undergo autopolymerization in vapor states. The monomers also undergo polymerization induced by free radicals. Successful inhibition of polymerization of alkyl-2-cyanoacrylates depends on the ability of the inhibitor to retard polymerization reaction (Ramotowski 2012). Figure 3 presents the polymerization of ethyl cyanoacrylate in the presence of water (weak base). The reaction of cyanoacrylates with latent print residue is considerably more complicated and less well understood. Variety of initiator compounds includes lactic acid, ammonia, acetic acid, amines, alcohols, amino acids, alkanes and proteins (Czekanski et al. 2006; Wargacki et al. 2007; Wargacki et al. 2008).

## Methodology

Cyanoacrylate, fuming tank, cabinet or other suitable container with a proper ventilation system are the equipment and materials required for processing articles bearing latent fingermarks (Ramotowski 2012).

### Test procedure

Latent fingerprints can be developed on and non-porous surfaces by following sequence of steps:

- Place the articles bearing latent fingermarks into the cabinet. Their surfaces should be exposed to the cyanoacrylate fumes.
- Place few drops of liquid cyanoacrylate into a small porcelain dish and place the dish into the fuming cabinet.
- Allow the items to be exposed to the fumes until whitish-colored fingerprint patterns appear (Ramotowski 2012).

Different methods have been reported for the processing of developing latent fingermarks using cyanoacrylate fuming. Simple homemade system consisting of a chamber, such as a glass aquarium, can be used to develop cyanoacrylate vapors by suitable heat source like hot plate, heater etc. and fumed the articles bearing latent impression at minimal cost. Articles to be processed are suspended in chamber and small amount of liquid cyanoacrylate is heated to around 80 to 100 °C to produce sufficient vapor. A container of water must also be placed in the tank to provide sufficient humidity for the development of prints. Low humidity causes the appearance of weakly developed prints with poor contrast. Development process should be regularly inspected to avoid over-development. Temperature control, proper vapor circulation and automatic removal of the cyanoacrylate vapor are some of the added advantages of commercial fuming chamber over home-made chamber. Some systems also have humidity control (Champod et al. 2004). Stokes and Brennan (1995) developed a reliable, robust and easy to use free standing cabinet for the automatic development of latent fingermarks using cyanoacrylate fuming. Some authors used cyanoacrylate treated neutral filter paper to develop latent fingermarks on cadavers, living skin, currency, kraft paper, leather, wood and silk fabric. The neutral filter paper was brushed or sprayed with cyanoacrylate ester or cyanoacrylate ether solution allowed to dry for several minutes. The treated paper was then put on the surface bearing latent fingermarks. This paper should be left on the surface for 5 to 60 min depending upon the nature of surface (Jian and Dao-an 1991). Almog and Gabay (1986) used glass desiccators and polythene bag as a fuming cabinet to develop latent fingermarks on different kinds of porous surfaces using cyanoacrylate fuming method.

It was observed that relative humidity and long term storage of latent fingermarks prior to processing had negligible effect on the quality of cyanoacrylate developed fingerprints on polypropylene foils (Schwarz and Hermanowski 2012). In contrast to this, other authors observed that relative humidity during cyanoacrylate fuming effects the quality of developed prints and microstructure of the polycyanoacrylate. However, relative humidity has less influence on sebaceous marks (Paine et al. 2011). Kendall observed that several hours to several days may take place to develop latent fingermarks using super glue fuming (Kendall 1982). However, heating of cyanoacrylate reduces the time required to develop latent fingermarks (Olenik 1984). Overheating of cyanoacrylate ester should be avoided as it produces toxic hydrogen cyanide, if heated above approximately 220 °C (Mock 1985; Masters 2002). Kendall and Rehn (1983) observed that polymerization of cyanoacrylate reduces the time required to develop latent fingermarks. Pires and Springer (2016) observed that aluminum did not retard the polymerization of cyanoacrylate prior to vaporization. However, in contrast to this, some studies reported that aluminum acts as a polymerization retardant in developing latent fingermarks using cyanoacrylate fuming method (Olenik 1984; Olenik 1983; Lewis et al. 2001; Tuthill 1982).

## Evaluation of cyanoacrylate fuming method

Bentolila et al. (2013) synthesized and used fluorescent monomers in cyanoacrylate fuming method for developing latent fingermarks on glass surface. Casault et al. (2017) compared different cyanoacrylates (methyl, ethyl, n-butyl and 2-octyl) for developing latent fingermarks on glass, plastic and metal surfaces. The use of methyl cyanoacrylate for the development of fresh and aged latent fingermarks on glass and plastic while n-butyl cyanoacrylate for plastic surface was recommended. They observed that composition of latent fingermark residue effect the polymer structure. Tahtouh et al. (2011) compared different alkyl 2-cyanoacrylate monomers (alkyl = 1-cyanoethyl, 2-cyanoethyl, trideuteromethyl and pentadeuteroethyl) for the development of latent fingermarks on wide range of semi-porous and non-porous surfaces and observed that 1-cyanoethyl 2-cyanoacrylate monomer gave the best results even on difficult surfaces like white opaque and fluorescent acrylic sheets and polymer banknotes printed with intaglio ink.

Cyanoacrylate fuming method was used to develop latent fingerprints on bullet casings by Babin (2010). Fieldhouse (2011) suggested the use of cyanoacrylate fuming method for developing both fresh and aged latent fingerprints on smooth and textured plastics. Amata et al. (2015) reported a case in which cyanoacrylate fuming method was used to develop latent fingermarks on the trigger of firearm. In another study, the use of cyanoacrylate fuming method was suggested to develop latent fingermarks on the duct tape (Olenik 1984). Tissier et al. (1999) reported a case in which latent fingermarks were developed inside a vehicle by cyanoacrylate fuming method. Vapors of cyanoacrylate were fed into car through a hose and car itself acts as a fumigation chamber. Matthias (2016) used cyanoacrylate fuming method to develop latent fingermarks on both adhesive and non-adhesive sides of different kinds of tapes (black electrical tape, packaging tape, grey duct tape, masking tape) which were previously treated with Un-Du liquid. It was observed that use of Un-Du liquid did not affect the quality of developed prints and also did not interfere in processing of items with cyanoacrylate fuming method. Lam et al. (2014) recommended the sequential processing with cyanoacrylate fuming, vacuum metal deposition and fluorescent dye staining to develop latent fingermarks on Canadian polymer banknotes. They suggested the immediate processing of banknotes with cyanoacrylate fuming in order to minimize the print degradation. In another study, Lam (2014) recommended the use of oblique light to examine and photograph cyanoacrylate developed fingerprints on Canadian polymer banknotes.

Trapecar and Balazic (2007) observed no significant difference in quality of developed prints on dead and live human skin by cyanoacrylate fuming method. However, they suggested the use of Swedish black powder and Ruthenium Tetroxide (RTX) over cyanoacrylate fuming method for developing latent fingermarks on live and dead skin. Midkiff and Codell (1995) observed that thickness, type and consistency of adhesive affect the quality of cyanoacrylate-fumed developed fingermarks. A novel luminescent cyanoacrylate such as lumicyano was used to develop latent fingerprints on glass and a number of semiporous and nonporous surfaces in a single processing step without any further treatment (Prete et al. 2013). One-step fluorescent cyanoacrylate fuming method was used to develop high-quality fluorescent prints on a wide range of nonporous surfaces including trash bags, sandwich bags, sheet protectors, bubble wrap and textured plastic. Authors observed that one-step fluorescent cyanoacrylate fuming method was more sensitive towards sebaceous prints than eccrine prints due to presence of high concentration of initiators in the former one (Hahn and Ramotowski 2012).

### Pre-treatment procedures

With the passage of time, natural dehydration occurs in latent fingermark deposit. Development of these latent fingermarks is difficult due to their exposure to harsh environmental conditions such as low humidity, UV light, or heat. Several pretreatment methods intended to reintroduce moisture to dehydrated fingermarks have been reported. These methods include exposure to acetic acid vapor, ammonia vapor and heated water vapor. Pre-exposure to UV and X-ray radiations was suggested for developing age-degraded latent fingermarks on glass by cyanoacrylate fuming method (Ristova et al. 2016). Pretreatment with vapors of ammonia (a weak base) was also suggested to reintroduce the moisture in dry latent fingermarks and thus improve the results of cyanoacrylate fuming on aged latent fingermarks (McLaren et al. 2010; Burns et al. 1998). Pre-treatment with valine-based and red fluorescent powders were suggested to develop aged latent fingerprints on black polyvinyl chloride by cyanoacrylate fuming method. It was observed that degradation of latent fingermarks depends on moisture (Nixon et al. 2013). The use of 10% *w/v* methylamine solution over dimethylamine, ethylamine and trimethylamine was suggested as an effective pretreatment solution to develop dry, depleted and aged latent fingermarks on a wide range of surfaces including polyethylene. They observed that pretreatment of aged latent fingermarks with 10% *w/v* methylamine solution improves the polymerization of cyanoacrylate (McLaren et al. 2010; Montgomery et al. 2012). Steele et al. (2012) observed that pre-cooling of non-porous (glass, copper, zinc-steel alloy) items prior to cyanoacrylate fuming significantly increases the detection sensitivity of developed prints due to the increased deposition of cyanoacrylate on latent fingermark residues which resulted due to modification in pseudo-crystalline structure of polymer. The process also increases the adherence of dye staining during post-treatment of cyanoacrylate developed fingerprints. Bouwmeester et al. (2016) suggested that lumicyano can be used in place of conventional cyanoacrylate as a preparatory (or pretreatment) step to develop latent fingermarks in blood on black polypropene sheet with SPR- W (small particle reagent white) and acid yellow 7. They observed that different processing methods did not affect the DNA recovery. Photography of developed fingermarks on the same day of their development was also advised.

### Post-treatment procedures

The cyanoacrylate-developed print may be further enhanced by dusting with regular or magnetic fingerprint powder (Ramotowski 2012). The cyanoacrylate developed fingerprints can further be enhanced by using Rhodamine 6G (Jian and Dao-an 1991). Morris (1992) suggested the use of basic yellow 40 in place of gentian violet for enhancing cyanoacrylate-fumed fingermarks on adhesive side of tape. Isaac (1993) used rhodamine 6G for enhancing cyanoacrylate-fumed fingermarks on different kinds of self-adhesive tapes. Kobus et al. (1983) suggested the use of gentian violet and coumarin 540 to enhance the quality of cyanoacrylate developed prints on polyethylene and aluminium foil respectively. Chesher et al. (1992) used acid fuchsin, Nile blue A, safranin bluish and Rit® fabric dye in combination with OMNIPRINT^TM^ 1000 alternate light source to enhance the quality of cyanoacrylate developed prints on white polyethylene dinner plate. Post-treatment with vapors of *p*-dimethylaminobenzaldehyde was suggested to improve the quality of cyanoacrylate-fumed latent fingerprints on solvent-sensitive surfaces such as oil marker writings and materials with rough surfaces such as unglazed earthenware (Takatsu et al. 2012). The use of RAY (Rhodamine, Ardrox, Basic Yellow) dye stain, gentian violet and alternate powder was suggested for enhancing the quality of cyanoacrylate-developed fingerprints on the adhesive side of various kinds of tapes. Processing of cyanoacrylate fumed prints with gentian violet, followed by alternate powder and finally with RAY dye stain was suggested as best processing sequence to enhance the quality of developed prints (Wilson 2010). Chadwick et al. (2014) observed that staining of Polycyano UV processed marks with rhodamine 6G significantly improves its performance on aluminium, glass and polyethylene bags. The use of sublimating dyes such as 1-amino-2-phenoxy-4-hydroxy-anthraquinone and 1,4-bis-(ethylamino)-anthraquinone was suggested to enhance the quality of cyanoacrylate developed (fresh and aged upto 2 months) prints on plastics, metals and glass surfaces. The particles of dyes adhere to polymerized cyanoacrylate and provide red purple or blue color to it (Morimoto et al. 1998). Lock et al. (1995) suggested the use of europium thenoyltrifluoroacetone ortho-phenanthroline (EuTTAPhen) complex for enhancing the quality of cyanoacrylate-developed prints. Olenik (2015) described a procedure to develop latent fingermarks on smooth and adhesive sides of duct tape and other plastic tapes, including black electrical tape using cyanoacrylate fuming followed by dyeing with basic yellow 40. The procedure involves the fuming of tape with cyanoacrylate vapors followed by application of basic yellow 40. After dyeing, tape is rinsed under running tap water, followed by drying and visualized with orange or yellow goggles under blue light as well as under forensic light source in the range of 415 to 485 nm. Menzel et al. (1983) observed that application of post-treatment procedures (dusting, staining and ninhydrin/zinc chloride) to cyanoacrylate developed prints significantly improves the detectibility of latent prints. They suggested the use to ultraviolet and blue-green argon-ion laser together with fluorescent powder dusting, fluorescent dye staining and ninhydrin/zinc chloride post-processing of cyanoacrylate developed prints. Sonnex et al. (2016) used cyanoacrylate fuming followed by Infrared spectral mapping for visualization and enhancement of latent fingermarks on smooth, shiny fabrics such as silk, nylon and polyester, of different colors and patterns. However, due to the presence of carbonyl functional group in cotton and polycotton and their absorbency to latent fingermark residues, these fabrics gave poor results. Stoltzfus and Rebane (2016) used NIR two-photon induced fluorescence imaging technique to image cyanoacrylate processed fingermarks on highly-reflective substrates. Chadwick et al. (2011) suggested the use of styryl 11, a laser dye, over rhodamine 6G for the enhancement of cyanoacrylate-developed fingermarks in near-infrared region. The luminescence emission intensity of STaR 11, combination of styryl 11 and rhodamine 6G, was better than styryl 11 and rhodamine 6G individually and could be used for enhancing the visualization of cyanoacrylate-developed fingermarks on multicolored glossy cardboard in visible and near-infrared regions.

### Comparative studies

Fraser et al. (2014) suggested the use of vaccum metal deposition (gold and zinc) over cyanoacrylate fuming method for developing latent fingermarks on different kinds of fabrics such as nylon, polyester, cotton and polycotton as it produced greater ridge detail and more effective than latter one. Dominick and Laing (2011) suggested the use of cyanoacrylate fuming followed by gun blue followed by brilliant yellow 40 dye staining and cyanoacrylate fuming followed by palladium deposition over cyanoacrylate fuming followed by brilliant yellow 40 dye staining, cyanoacrylate fuming followed by gun blue, powder suspension and palladium suspension for developing latent fingermarks on unfired brass cartridge cases. They suggested that incorporation of a specific metal treatment into well established reported nonporous fingermark enhancement technique increases the yield of potentially identifiable fingermarks.

Farrugia et al. (2015) suggested the use of atmospheric/humidity fuming process over vacuum fuming process for both two-step and one-step cyanoacrylate fuming for developing latent fingermarks on plastic carrier bags. They also compared two-step cyanoacrylate fuming using basic yellow 40 (LCA4% → LCA4% → BY40) and a one-step fluorescent cyanoacrylate fuming using Lumicyano and observed that two-step process gave better quality prints than one-step process under vacuum conditions. They also observed that increase in amount of Lumicyano 4% cyanoacrylate and fuming time gave lower detection rate than double process with Lumicyano 4%. However, double process with conventional cyanoacrylate did not improve the detection rate. In another study, Farrugia et al. (2014a) advocate the use of Lumicyano™ (one-step fluorescent cyanoacrylate product) followed by basic yellow 40 dyeing (LCA1% → BY40) over cyanoacrylate fuming followed by basic yellow 40 dyeing (CA → BY40) and powder suspensions for developing latent fingermarks on plastic carrier bags as its use is easy, safe and fast requiring no drying or dyeing process without compromising the quality of developed prints. Farrugia et al. (2014b) suggested the use of Lumicyano solution (LCA solution → BY40) and Lumicyano powder (LCA powder → BY40) for developing latent fingermarks on different kinds of semiporous substrates such as, junk mail, magazines, cardboard packaging. Four percent Lumicyano → BY40 in place of solvent black 3 and iron-oxide powder suspensions was also suggested to develop greasy fingermarks on semiporous surfaces. Payne et al. (2014) observed that sebaceous prints on bass surface degrade more quickly in light than in dark. They suggested that silver electroless deposition (SED) is a suitable alternative method to cyanoacrylate fuming method for development of latent fingermarks on brass surfaces except for metals which are coated in a lacquer. Snyder (2009) suggested the use of Sudan black over magnetic powder, ninhydrin, physical developer, cyanoacrylate with Sudan black, cyanoacrylate with ninhydrin and cyanoacrylate with physical developer to develop latent fingermarks in petroleum jelly. Boateng et al. (2016) used ABC dry fire extinguisher powder to develop latent fingermarks on heated and unheated tile, metal and glass surfaces. They compared fire extinguisher powder method with fluorescent powder, magnetic powder, granular white or black powder and cyanoacrylate → rhodamine methods and observed that quality of prints developed with fire extinguisher powder is comparable to prints developed by these methods on both heated and unheated surfaces. They suggested that fire extinguisher powder binds to metal ions present in latent fingermark residues.

Edmiston and Johnson (2009) suggested the use of cyanoacrylate → powder → acidified hydrogen peroxide → rhodamine 6G sequence for developing latent fingermarks on brass casings. However, the sequence consisting of cyanoacrylate → rhodamine 6G → acidified hydrogen peroxide → powder was suggested to develop latent fingermarks on nickel casings and shotgun shells. Girelli et al. (2015) observed that cyanoacrylate fuming, gun blue and basic yellow 40 gave better quality prints than powder dusting (regular and magnetic powder) and acidified hydrogen peroxide solutions. They suggested the sequential application of cyanoacrylate, gun blue and basic yellow 40 for developing latent fingermarks on fired and unfired cartridge cases. They also observed that firing process significantly effects amount of latent deposit and thereby quality of developed prints.

Scott (2009) observed that treatment of adhesive side of some items (latex and nitrile gloves, duct, masking and scotch tape) with cyanoacrylate fuming causes interference with subsequent powder suspension processing. He suggested that these items should not be processed with cyanoacrylate fumes before powder suspension processing. Pleckaitis (2007) observed that treatment of inner surface of latex and nitrile gloves with cyanoacrylate fumes causes interferences with subsequent further processing.

### Effect of cyanoacrylate fuming on the detection of drugs of abuse and composition of latent fingermark residues

Day et al. (2004) observed that cyanoacrylate fuming process did not cause any interference in the detection of drugs of abuse (codeine phosphate, cocaine hydrochloride, amphetamine sulphate, barbital and nitrazepam) and potential adulterants (caffeine, aspirin, paracetamol, starch and talc) by Raman spectroscopy. Raman bands due to cyanoacrylate polymer were present but their presence did not cause any difficulty in establishing the identity of these drugs in even in the presence of adulterants. Sundar and Rowell (2014) identify drugs of abuse (cocaine and methadone) and therapeutic drugs (aspirin, caffeine and paracetamol) by Surface Assisted Laser Desorption Ionisation Time of Flight Mass Spectrometry (SALDI-TOFMS) or Matrix Assisted Laser Desorption Ionisation TOF-MS (MALDI-TOF-MS). They observed that exposure of cyanoacrylate developed prints to acetone vapor makes physical transfer of prints to lifting tape in easy and simple manner. Koenig et al. (2011) observed no significant effect of cyanoacrylate fuming method on the initial composition of latent fingermark residues.

### Effect of temperature on cyanoacrylate fuming

Deans (2006) used cyanoacrylate fuming method to develop latent fingermarks on items exposed to temperatures of around 500 °C. Gardner et al. (2016) suggested the use of black magnetic powder and cyanoacrylate fuming followed by Brilliant Yellow 40 staining to develop latent fingermarks on items recovered from fire. Sanders (2011) observed that cyanoacrylate fuming method is less effective in developing latent fingermarks on explosive devices containing smokeless powder.

### Effect of water on cyanoacrylate fuming

Soltyszewski et al. (2007) suggested the use of ferromagnetic powder and cyanoacrylate fuming methods over aluminium powder to develop fresh and aged (up to six weeks) latent fingermarks on glass recovered from water. Trapecar (2012) suggested the use of cyanoacrylate fuming over silver special powder and small particle reagent (black and white) to develop fresh and aged (168 h old) latent fingermarks on glass and burnished metal surfaces submerged in stagnant water.

### Effect of cyanoacrylate fuming on the DNA analysis

Bille et al. (2009) observed that cyanoacrylate fuming did not cause any interference in recovery and subsequent DNA analysis from biological materials extracted from fragments of post blast pipe bomb. However, cyanoacrylate fuming significantly effects the amplification of DNA depending on the extraction methods. The effect of cyanoacrylate fuming on genetic typing is negligible when DNA is extracted using Invisorb kit. It also affects the PCR products (Wurmb et al. 2001). Bhoelai et al. (2011) recovered a partial DNA profile after processing latent fingermarks with cyanoacrylate fuming. Shipp et al. (1993) reported that cyanoacrylate fuming had no effect on either the amount or patterning of DNA extracted from human bloodstains.

## Conclusion

Cyanoacrylate fuming is a chemical method for development of latent fingermarks on non-porous surfaces. Although it is a time consuming and laborious method yet its versatility and efficiency towards developing latent fingermarks on wide range of non-porous surfaces cannot be ignored. It develops stable white colored fingerprints. The quality of developed prints can be improved by subsequent powdering and/or staining procedures. It is an efficient, non-destrcutive and excellent procedure for developing latent fingermarks.
